# A phase II study of sequential chemotherapy with docetaxel after the weekly PELF regimen in advanced gastric cancer. A report from the Italian group for the study of digestive tract cancer

**DOI:** 10.1054/bjoc.2000.1631

**Published:** 2001-02

**Authors:** S Cascinu, F Graziano, S Barni, R Labianca, G Comella, R Casaretti, L Frontini, V Catalano, A M Baldelli, G Catalano

**Affiliations:** Division of Medical Oncology, Azienda Ospedale di Parma, Italy; Medical Oncology Unit, Azienda Ospedale di Urbino, Italy; Division of Medical Oncology, Azienda Ospedale di Treviglio, Italy; Medical Oncology Unit, Azienda Ospedale di Bergamo, Italy; National Tumor Institute, Naples, Italy; Medical Oncology Unit, Ospedale San Paolo, Milan, Italy; Division of Medical Oncology, Azienda Ospedale di Pesaro, Italy

**Keywords:** gastric cancer, sequential chemotherapy, docetaxel

## Abstract

In advanced gastric cancer, we investigated feasibility and activity of sequential chemotherapy with docetaxel after an intensive weekly regimen consisting of cisplatin, epidoxorubicin, fluorouracil, leucovorin (PELF) plus filgrastim. Chemotherapy-naive patients with relapsed or metastatic gastric cancer received 8 weekly administrations of chemotherapy with cisplatin 40 mg/m^2^, fluorouracil 500 mg/m^2^,epi-doxorubicin 35 mg/m^2^, 6S-steroisomer of leucovorin 250 mg/m^2^and glutathione 1.5 g/m^2^. On the other days filgrastim 5 μg kg^–1^was administered by subcutanous injection. Subsequently, patients with partial response or stable disease received 3 cycles of docetaxel 100 mg/m^2^every 3 weeks. 40 patients have been enrolled and they are evaluable for response and toxicity. After the PELF regimen, 3 patients achieved complete response, 13 patients showed partial response, 21 patients had stable disease and 3 patients progressed (40% response rate; 95% CI 25% to 55%). After docetaxel, 9 out 34 patients improved the outcome (26.5%); 7 patients with stable disease achieved partial response and 2 patients with partial response achieved complete response. The overall response rate in the 40 patients was 57.5% (95% CI, 42.5% to 72.5%). The PELF regimen did not cause any grade IV toxicity, the most frequent grade III acute side-effects were thrombocytopenia and vomiting which occurred in the 10% of 320 PELF cycles. Docetaxel caused grade III–IV neutropenia and thrombocytopenia in the 10% and the 19% of cycles respectively. Fatigue was a frequent side-effect during both PELF and docetaxel chemotherapy. The sequential application of docetaxel after PELF chemotherapy gained major objective responses with manageable toxicity. This strategy is worth of further investigation in the setting of palliative or neoadjuvant chemotherapy. © 2001 Cancer Research Campaign http://www.bjcancer.com


*British Journal of Cancer* (2001) **84**(4), 470–474
© 2001 Cancer Research Campaign
doi: 10.1054/ bjoc.2000.1631, available online at http://www.idealibrary.com on IDEAL®

http://www.bjcancer.com

### A phase II study of sequential chemotherapy with docetaxel after the weekly PELF regimen in advanced gastric cancer. A report from the Italian group for the study of digestive tract cancer

**S Cascinu[1], F Graziano[2], S Barni[3], R Labianca[4], G Comella[5], R Casaretti[5], L Frontini[6], V Catalano[7], AM Baldelli[7] and G Catalano[7]**

[1]Division of Medical Oncology, Azienda Ospedale di Parma, Italy; [2]Medical Oncology Unit, Azienda Ospedale di Urbino, Italy; [3]Division of Medical Oncology, Azienda Ospedale di Treviglio, Italy; [4]Medical Oncology Unit, Azienda Ospedale di Bergamo, Italy; [5]National Tumor Institute, Naples, Italy; [6]Medical Oncology Unit, Ospedale San Paolo, Milan, Italy; [7]Division of Medical Oncology, Azienda Ospedale di Pesaro, Italy

**Summary** In advanced gastric cancer, we investigated feasibility and activity of sequential chemotherapy with docetaxel after an intensive weekly regimen consisting of cisplatin, epidoxorubicin, fluorouracil, leucovorin (PELF) plus filgrastim. Chemotherapy-naive patients with relapsed or metastatic gastric cancer received 8 weekly administrations of chemotherapy with cisplatin 40 mg/m$^2$, fluorouracil 500 mg/m$^2$,epidoxorubicin 35 mg/m$^2$, 6S-steroisomer of leucovorin 250 mg/m$^2$ and glutathione 1.5 g/m$^2$. On the other days filgrastim 5 µg kg$^{-1}$ was administered by subcutanous injection. Subsequently, patients with partial response or stable disease received 3 cycles of docetaxel 100 mg/m$^2$ every 3 weeks. 40 patients have been enrolled and they are evaluable for response and toxicity. After the PELF regimen, 3 patients achieved complete response, 13 patients showed partial response, 21 patients had stable disease and 3 patients progressed (40% response rate; 95% CI 25% to 55%). After docetaxel, 9 out 34 patients improved the outcome (26.5%); 7 patients with stable disease achieved partial response and 2 patients with partial response achieved complete response. The overall response rate in the 40 patients was 57.5% (95% CI, 42.5% to 72.5%). The PELF regimen did not cause any grade IV toxicity, the most frequent grade III acute side-effects were thrombocytopenia and vomiting which occurred in the 10% of 320 PELF cycles. Docetaxel caused grade III–IV neutropenia and thrombocytopenia in the 10% and the 19% of cycles respectively. Fatigue was a frequent side-effect during both PELF and docetaxel chemotherapy. The sequential application of docetaxel after PELF chemotherapy gained major objective responses with manageable toxicity. This strategy is worth of further investigation in the setting of palliative or neoadjuvant chemotherapy. © 2001 Cancer Research Campaign http://www.bjcancer.com

**Keywords**: gastric cancer; sequential chemotherapy; docetaxel

Gastric cancer is considered a chemosensitive disease and second generation combination chemotherapy regimens have produced high response rates and impressive survival times (Hill and Cunningham, 1998). Unfortunately, about half of the patients treated with chemotherapy is unresponsive, and less than half of the patients with locally advanced disease is amenable of surgical resection after neoadjuvant chemotherapy. For these reasons, new and hopefully more effective drugs, or innovative treatment strategies are needed.

Docetaxel is a semisynthetic taxoid with cytotoxic activity against a broad spectrum of human solid tumors (Cortes and Pazdur, 1995). Docetaxel has been tested in advanced gastric cancer and it showed promising single-agent activity with 20% to 24% response rates in treated and chemotherapy-naive patients (Sulkes et al, 1994; Furue and Taguchi, 1998; Mavroudis et al, 1999; Vanhofer et al, 1999). A logical step of investigation consisted in the development of multi-drug schedules including docetaxel and other known active drugs. In early phase II studies, combinations of docetaxel with cisplatin, fluorouracil or epidoxorubicin showed promising results, but neutropenia and non-haematologic toxicity were often significant (Ajani et al, 1999; Andrè et al, 1999; Roth et al, 2000).

Ongoing studies are exploring new schedules of docetaxel-based combination chemotherapy to ameliorate the efficacy/toxicity ratio. Sequential schedules may maximize the dose-intensity of each single agent and avoid the overlapping toxicity caused by the concomitant administration of active drugs. Safety and efficacy of sequential chemotherapy with docetaxel has been tested in breast cancer with favourable results (Antoine et al, 1998), and this chemotherapeutic strategy deserves investigation in other tumors with documented activity of docetaxel (Pronk et al, 1995).

In advanced gastric cancer, we investigated safety and activity of sequential chemotherapy with docetaxel after the intensive weekly PELF regimen (Cascinu et al, 1997, 1998).

### MATERIALS AND METHODS

### Patient selection

Chemotherapy-naive patients with pathologically confirmed, relapsed or metastatic gastric cancer were considered eligible for

*Received 27 July 2000*
*Revised 1 November 2000*
*Accepted 21 November 2000*

*Correspondence to*: S Cascinu the study. Other eligibility criteria were: Eastern Cooperative Oncology Group (ECOG) performance status 0, 1, or 2; age equal or less than 75 years; normal liver, renal, and bone marrow functions. The protocol was approved by each local institutional review board and all patients gave written informed consent.

### Treatment plan

PELF chemotherapy consisted of a 1 day per week administration of cisplatin 40 mg/m$^2$, fluorouracil 500 mg/m$^2$, epi-doxorubicin 35 mg/m$^2$, 6S-stereoisomer of leucovorin 250 mg/m$^2$ and glutathione 1.5 g/m$^2$. All drugs were given intravenously and on the other days filgrastim was administered by subcutanous injection at a dose of 5 μg kg$^{-1}$ (Cascinu et al, 1997). After 8 weekly cycles patients were re-evaluated and those with partial response or stable disease received docetaxel 100 mg/m$^2$ via a 1-hour intravenous infusion every 3 weeks. After 3 cycles with docetaxel patients were re-evaluated for response to the sequential treatment. All patients received emesis prophylaxis with 5-HT3 inhibitors and hyperhydration during each course of PELF chemotherapy. Patients who received docetaxel were treated with dexamethasone 8 mg p.o. administered 12 and 6 hours before drug infusion and 8 mg twice daily for an additional 4 days.

Full doses of the anticancer drugs were given if the neutrophil count was equal or $>1.5 \times 10^9$ l$^{-1}$ and the platelet count equal or $>100 \times 10^9$ l$^{-1}$; dose reductions were not recommended and values less than these necessitated a 7-day treatment delay. Patients treated with docetaxel did not receive prophylactic haematopoietic growth factors. However, filgrastim was employed in patients with grade III neutropenia lasting more than one week or grade IV neutropenia, so that treatment at the 100 mg/m$^2$ dose level could be maintained.

### Evaluation procedures

Pretreatment evaluation consisted of baseline studies including: medical history, physical examination, blood chemistries, urinoanalysis and ECG. Also, chest X-rays, abdominal computed tomography or magnetic resonance, bone scan and any other test to identify the extent of disease was performed. These studies were repeated after 8 weekly administration of PELF chemotherapy, after 3 cycles of docetaxel and every 3 months thereafter. Responses to the sequential program were not confirmed by an early repeat estimation.

All patients had physical examination and biochemical profile before each administration of chemotherapy. Response and toxicity were evaluated and graduated according to the standard World Health Organization (WHO) criteria (Miller et al, 1981). Patients treated with docetaxel with no fluid retention were considered grade 0; asymptomatic weight gain, grade 1; mild peripheral oedema that did not require diuretics, grade 2; symptomatic, moderate edema tha required diuretics, grade 3; edema/fluid retention that necessitated docetaxel withdrawal, grade 4.

### Statistical plan

The optimal two-stage design was adopted for this phase II trial (Simon, 1989). The minimum target activity level was a 20% gain in objective responses attained by docetaxel after the PELF regimen. Early discontinuation of the study was planned in the case of no response in the first 12 assessable patients treated with docetaxel ($\alpha$ and $\beta$ error probabilities 0.010 and 0.010). Alternatively, a planned sample size of approximately 30 patients was chosen to better estimate efficacy; 35% maximum width of the 95% confidence interval (CI) for the overall response rate. Time to disease progression was measured from date of registration to the date of progressive disease. Overall survival was measured form the time of registration to the date of death resulting from any cause.

### RESULTS

Between October 1998 and November 1999 40 patients entered this study and they are fully evaluable for response and toxicity. Their characteristics are reported in Table 1.

The toxicity profile of the PELF regimen was acceptable and it was similar to that of previous studies. None of the 40 patients suffered from grade IV toxicity and 12 patients (35.3%) experienced acute grade III adverse events. The major grade III toxicities were thrombocytopenia and vomiting which occurred in the 10% of 320 PELF cycles (Table 2). The most frequent chronic adverse events attributable to the PELF regimen (Table 3) were grade III alopecia in the 88% of the patients and grade II asthenia in the 25% of the patients. Transient grade II peripheral neuropathy occurred in 2 patients. All the 40 patients received eight cycles, but due to neutropenia and/or thrombocytopenia, the 30% of 320 PELF administrations were delayed a week. After the PELF regimen, 3 patients achieved complete response, 13 patients showed partial response, 21 patients had stable disease and 3 patients progressed (40% response rate; 95% CI 25% to 55%).

According to the treatment protocol 34 patients started docetaxel and all of them completed 3 cycles of chemotherapy. Acute adverse events in 102 cycles are listed in Table 4; grade III–IV neutropenia and thrombocytopenia occurred in the 10% and the 19% of cycles, respectively. None of the patients experienced neutropenic fever or sepsis, but 10 patients with grade III/IV neutropenia were treated with prophylactic filgrastim to maintain the planned dose of chemotherapy. Due to neutropenia and/or thrombocytopenia, docetaxel was delayed a week in the 45% of 102 cycles. One patient with grade III dermatitis had 50% dose reduction in the last administration of docetaxel. Asthenia was the most frequent chronic adverse events (Table 5) and it resulted grade I/II in 18 patients and grade III in 2 patients. None of the

**Table 1** Characteristics of the 40 patients enrolled in the study

| | |
|---|---|
| Number of patients | 40 |
| Sex ratio M/F | 23/17 |
| Median age (range) | 57 y (38–69) |
| ECOG performance status | |
| 0 | 8 |
| I | 22 |
| II | 10 |
| Prior surgery | |
| None | 5 |
| Curative | 22 |
| Palliative | 13 |
| Disease sites: | |
| Liver | 14 |
| Lymph nodes + abdominal mass | 13 |
| Liver + lymph nodes | 5 |
| Local relapse | 5 |
| Lung + liver | 2 |
| Lung | 1 |

*© 2001 Cancer Research Campaign*

**Table 2** Acute adverse events associated with the PELF regimen in 320 cycles

| Toxicity | WHO Grade 0 | 1 | 2 | 3 |
|---|---|---|---|---|
| | No of cycles (%) | No of cycles (%) | No of cycles (%) | No of cycles (%) |
| Neutropenia | 186 (58) | 76 (24) | 38 (12) | 20 (6) |
| Thrombocytopenia | 177 (55) | 50 (15) | 63 (20) | 30 (10) |
| Anaemia | 189 (59) | 70 (21) | 40 (12) | 21 (8) |
| Nausea/vomiting | 140 (43) | 99 (31) | 50 (16) | 31 (10) |
| Diarrhoea | 200 (62) | 79 (24) | 41 (14) | 0 |
| Mucositis | 274 (85) | 33 (11) | 13 (4) | 0 |

None of the patients treated with the PELF regimen experienced grade 4 side-effects.

**Table 3** Chronic adverse events associated with the PELF regimen in 40 enrolled patients

| Toxicity | WHO Grade 0 | 1 | 2 | 3 |
|---|---|---|---|---|
| | No of pts (%) | No of pts (%) | No of pts (%) | No of pts (%) |
| Asthenia | 22 (55) | 8 (20) | 10 (25) | 0 |
| Peripheral neurotoxicity | 32 (80) | 6 (15) | 2 (5) | 0 |
| Nail toxicity | 33 (82) | 4 (10) | 3 (8) | 0 |
| Constipation | 20 (50) | 20 (50) | 0 | 0 |
| Alopecia | 0 | 0 | 5 (12) | 35 (88) |

None of the patients treated with the PELF regimen experienced grade 4 side-effects.

**Table 4** Acute adverse events attributable to docetaxel in 102 cycles

| Toxicity | WHO Grade 0 | 1 | 2 | 3 | 4 |
|---|---|---|---|---|---|
| | No of cycles (%) | No of cycles (%) | No of cycles (%) | No of cycles (%) | No of cycles (%) |
| Neutropenia | 20 (19) | 37 (36) | 35 (35) | 9 (9) | 1 (1) |
| Thrombocytopenia | 18 (17) | 42 (41) | 23 (23) | 19 (19) | 0 |
| Anaemia | 48 (47) | 40 (39) | 14 (14) | 0 | 0 |
| Nausea/vomiting | 12 (12) | 44 (43) | 31 (30) | 15 (15) | 0 |
| Diarrhoea | 68 (67) | 22 (21) | 12 (12) | 0 | 0 |
| Mucositis | 87 (85) | 12 (12) | 3 (3) | 0 | 0 |
| Skin toxicity | 66 (65) | 24 (23) | 11 (11) | 1 (1) | 0 |
| Myalgias | 77 (75) | 13 (13) | 2 (2) | 0 | 0 |
| Dacryorrhoea | 68 (67) | 34 (33) | 0 | 0 | 0 |

**Table 5** Chronic adverse events attributable to docetaxel in 34 patients

| Toxicity | WHO Grade 0 | 1 | 2 | 3 |
|---|---|---|---|---|
| | No of pts (%) | No of pts (%) | No of pts (%) | No of pts (%) |
| Asthenia | 14 (42) | 8 (23) | 10 (29) | 2 (6) |
| Peripheral neurotoxicity | 28 (82) | 2 (6) | 4 (12) | 0 |
| Fluid retention | 29 (85) | 0 | 5 (15) | 0 |
| Constipation | 30 (88) | 4 (12) | 0 | 0 |
| Nail toxicity | 68 (67) | 22 (21) | 4 (12) | 0 |
| Alopecia | 0 | 0 | 2 (6) | 32 (94) |

Chronic grade 4 side-effects were not observed in patients receiving docetaxel.

 © 2001 Cancer Research Campaign patients experienced hypersensitivity reactions but moderate fluid retention syndrome was observed in 5 patients.

None of the 34 patients progressed during docetaxel chemotherapy and 9 of them (26.5%) gained a major response; 7 patients with stable disease improved to partial response and 2 patients with partial response achieved complete response. Excluding from the overall response rate the two patients who improved partial response to complete response, the PELF-docetaxel regimen produced unconfirmed objective responses in 23 out of 40 patients (57.5% response rate with 95% CI, 42.5% to 72.5%). Time to disease progression and median survival time resulted 7 months and 12.6 months, respectively.

### DISCUSSION

Recent phase II studies have established the role of docetaxel in first-line and second-line treatment of advanced gastric cancer (Sulkes et al, 1994; Furue and Taguchi, 1998; Mavroudis et al, 1999). As a consequence, the hope for more potent regimens prompted several investigators to evaluate docetaxel in multi-drug regimens. Early investigations of polychemotherapy with docetaxel, cisplatin and fluorouracil yelded high response rates, but side-effects were often pronounced due to overlapping toxicity (Andrè et al, 1999; Ajani et al, 2000; Roth et al, 2000). Roth et al (2000) reported a 56% response rate with the docetaxel-cisplatin combination. However, this schedule caused relevant haematologic toxicity with a high number of grade III/IV episodes of neutropenia (80% of the cycles) and non-fatal febrile neutropenia in 19% of the patients. Ajani et al (2000) treated patients with advanced gastric cancer with the combination of docetaxel, cisplatin and fluorouracil. The incidence of grade III/IV neutropenia was 72% per cycle. In addition, grade III/IV stomatitis was observed in the 16% of cycles. Available data suggest that about 20% to 30% of patients treated with these regimens do not receive the treatment on schedule for dose reductions or delays, and up to the 20% of patients discontinue therapy due to toxicity.

Combining old anti-cancer drugs with new compounds is a formidable challenge which requires several attempts to optimize the efficacy/toxicity ratio. Sequential chemotherapy (Day, 1986) and dose-dense schedules (Fizazi and Zelek, 2000) may offer this opportunity. In the present experience, patients received first-line chemotherapy with four of the most active drug in advanced gastric cancer, and the toxicity profile of the PELF-docetaxel chemotherapy seemed more favourable than that of new combinations using a concomitant administration of drugs. Interestingly, sequential docetaxel caused more episodes of grade II neutropenia (35% vs 12% of cycles) and grade III thrombocytopenia (19% vs 10% of cycles) than the PELF induction. Also, fatigue was more frequent after chemotherapy with docetaxel. According to our protocol, filgrastim was used after every cycle of the PELF regimen, whilst it was employed in the case of grade III–IV neutropenia during docetaxel chemotherapy. This may explain differences in the number of cycles with neutropenia between the PELF regimen and docetaxel chemotherapy. Also, it is possible that patients receiving the sequential programme were more likely to experience side-effects due to the prolonged exposure to chemotherapy with cumulative toxicity.

In the first phase II study (Cascinu et al, 1997), the PELF regimen showed 62% overall response rate which dropped to 46% in a subsequent analysis in patients with locally advanced disease (Cascinu et al 1998). In the present study, PELF chemotherapy alone achieved 40% response rate which approached 60% after docetaxel. The well-known ECF regimen showed 71% response rate in early phase II studies (Findlay et al, 1994) which dropped to 45% in a randomized trial (Webb et al, 1997). Phase III trials allows a proper analysis of response rates, survival, adverse events and quality of life and they are necessary for the testing of second-generation chemotherapy regimens in advanced gastric cancer. The non-randomized design of the PELF trials does not allow any definitive conclusion and any direct comparison for efficacy. The high response rate showed by the PELF chemotherapy in the early investigation needs to the confirmed in a randomized study, and a comparison with the PELF-docetaxel sequence would be of interest. At present, we may consider the PELF-docetaxel as an interesting evolution of the PELF regimen; this new sequential combination showed a favourable toxicity/efficacy ratio and it deserves further investigation in the palliative or neoadjuvant setting.

New combination chemotherapy regimens with substantial response rates and moderate toxicity may be studied as neoadjuvant chemotherapy (Kelsen, 1996). After PELF chemotherapy, 13 out of 32 patients with unresectable, locally advanced disease underwent surgery and their tumour was completely removed (Cascinu et al, 1998). Toxicity was acceptable, neither treatment-related deaths, nor surgical complications were observed. Also the ECF regimen was employed in the neoadjuvant setting (Findlay et al, 1994; Melcher et al, 1996). In these experiences, the ECF chemotherapy showed mild toxicity, and it allowed surgery in about half of patients with locally advanced disease.

An innovative strategy of neoadjuvant chemotherapy should consider the identification of patients with chemosensitive disease (Reichle et al, 2000). In fact, sequential chemotherapy may display the population of patients who may ulteriorly respond to non-cross resistant agents. In our previous experience with the PELF regimen (Cascinu et al, 1997), all patients but one achieved a maximum response after 8 cycles and 6 more cycles of the same chemotherapy did not improve the outcome. In the present study, docetaxel following PELF induction gained major responses, moreover, patients who improved after docetaxel had a major tumor shrinkage rather than a simple turn of minor response to partial response. This effect may be beneficial in a population of patients with locally advanced disease, by increasing the chance of successful surgical resection.

In conclusion, the discovery of new active compounds and their testing in multi-drug regimens has allowed progresses in the medical management of gastric cancer. Future trials will confirm or not the superiority of second-generation polychemotherapy regimens and their role in the palliative or the neoadjuvant setting. In this perspective, the PELF-docetaxel chemotherapy is worth of further investigations and we are planning a phase II analysis in neoadjuvant chemotherapy and a randomized trial of PELF vs PELF-docetaxel in metastatic disease.

### REFERENCES

Ajani JA, Fodor M, Van Cutsem E, Tjulandin S, Moiseyenko V, Cabral F, Majilis A, Chao Y, Zuber A, Blattmann C, Garay C and Jacques C (2000) Multinational randomized phase II trial of docetaxel and cisplatin with or without 5-fluorouracil in patients with advanced gastric cancer or GE junction adenocarcinoma. *Proc Am Soc Clin Oncol* **19**: 247

Andrè T, Louvet C, Ychou M, Gamelin E, Mousseau E, Carola S, Assadourian S and De Gramont A (1999) Docetaxel-epirubicin as second-line treatment for patients with advanced gastric cancer. *Proc Am Soc Clin Oncol* **18**: 277

© 2001 Cancer Research Campaign 

Antoine EC, Chollet P, Monfardini S, Sorio R, Ambrosini G, Benhammouda A, Mazen MF, Ramazeilles C, Azli N and Khayat D (1998) Sequential administration of docetaxel followed by AC in first-line metastatic breast cancer patients: final results. *Ann Oncol* **9** (4): 19

Cascinu S, Labianca R, Alessandroni P, Marcellini M, Silva RR, Pancera G, Testa E, Martignoni G, Barni S, Frontini L, Zaniboni A, Luporini G, Cellerino R and Catalano G (1997) Intensive weekly chemotherapy for advanced gastric cancer using fluorouracil, cisplatin, epidoxorubicin, 6S-leucovorin, gluthatione and filgrastim: a report of the Italian Group for the Study of the Digestive Tract Cancer. *J Clin Oncol* **15**: 3313–3319

Cascinu S, Labianca R, Graziano F, Pancera G, Barni S, Frontini L, Luporini G, Cellerino R and Catalano G (1998) Intensive weekly chemotherapy for locally advanced gastric cancer using 5-fluorouracil, cisplatin, epidoxorubicin, 6S-leucovorin, gluthatione and filgrastim: a report from the Italian Group for the Study of Digestive Tract Cancer (GISCAD). *Br J Cancer* **78**: 390–393

Cortes JE and Pazdur R (1995) Docetaxel. *J Clin Oncol* **13**: 2643–2655

Day RS (1986) Treatment sequencing, asymmetry and uncertainty: protocol strategies for combination chemotherapy. *Cancer Res* **46**: 3876–3885

Findlay M, Cunningham D, Norman A, Mansi J, Nicolson M, Hickish T, Nicolson V, Nash A, Sacks N and Ford H (1994) A phase II study in advanced gastro-esophageal cancer using epirubicin and cisplatin in combination with continuous infusion 5-fluorouracil (ECF). *Ann Oncol* **5**: 609–616

Fizazi K and Zelek L (2000) Is one cycle every three or four weeks obsolete? A critical review of dose-dense chemotherapy in solid neoplasms. *Ann Oncol* **11**: 133–149

Furue H and Taguchi T (1998) A late phase II study of RP56976 (docetaxel) in patients with advanced or recurrent gastric cancer. *Ann Oncol* **9** (supp.4): 49

Hill ME and Cunningham D (1998) Medical management of advanced gastric cancer. *Cancer Treat Rev* **24**: 113–118

Kelsen DP (1996) Adjuvant and neoadjuvant therapy for gastric cancer. *Semin Oncol* **23**: 379–389

Mavroudis D, Kakolyris S, Kouroussis Ch, Androulakis N, Agelaki S, Kalbakis K, Sarra E, Vardakis N, Souglakos J, Hatzidaki D, Malliotakis P, Samonis G and Geogoulias G (1999) First line treatment of advanced gastric cancer with docetaxel monotherapy and granulocyte colony-stimulating factor (G-CSF). *Proc Am Soc Clin Oncol* **18**: 254

Melcher AA, Mort D and Maughan TS (1996) Epirubicin, cisplatin and continuous infusion 5-fluorouracil (ECF) as neoadjuvant chemotherapy in gastro-oesophageal cancer. *Br J Cancer* **74**: 1651–1654

Miller AB, Hoogstraten B, Staquet M and Winkler V. Reporting results of cancer treatment (1981) *Cancer* **47**: 207–214

Pronk LC, Stoter G and Verweij J (1995) Docetaxel (Taxotere): single agent activity, development of combination treatment and reducing side-effects. *Cancer Treat Rev* **21**: 463–478

Reichle A, Jauch K, Hofstaedter F, Bataille F, Erdmann A, Kreuser E and Andreesen R (2000) Preoperative chemotherapy and consecutive gastrectomy in chemosensitive gastric cancer. Testing a new strategy. *Proc Am Soc Clin Oncol* **19**: 298

Roth AD, Maibach R, Martinelli G, Fazio N, Aapro MS, Pagani O, Morant R, Borner MM, Herrmann R, Honegger H, Cavalli F, Alberto P, Castiglione M and Goldhirsch A (2000) Docetaxel (Taxotere)-cisplatin (TC): an effective drug combination in gastric carcinoma. *Ann Oncol* **11**: 301–306

Simon R (1989). Optimal two-stage designs for phase II clinical trials. *Controlled Clin Trials* **10**: 1–10

Sulkes A, Smyth J, Sessa C, Dirix LY, Vermorken JB, Kaye S, Wanders J, Franklin H, LeBail N and Verweij J (1994) Docetaxel (Taxotere) in advanced gastric cancer: results of a phase II clinical trial. *Br J Cancer* **70**: 380–383

Vanhoefer U, Wilke H, Harstrick A, Achterrath W, Preusser P, Sthal M, Clemens MR, Thiel E, Flasshove M, Fink U, Trenn G and Seeber S (1999) Phase II study of docetaxel as second line chemotherapy in metastatic gastric cancer. *Proc Am Soc Clin Oncol* **18**: 303

Webb A, Cunningham D, Scarffe JH, Harper P, Norman A, Joffe JK, Hughes M, Mansi J, Findlay M, Hill A, Oates J, Nicolson M, Hickish T, O'Brien M, Iveson T, Watson M, Underhill C, Wardley A and Meehan M (1997) Randomized trial comparing epirubicin, cisplatin, and fluorouracil versus fluorouracil, doxorubicin, and methotrexate in advanced esophagogastric cancer. *J Clin Oncol* **15**: 261–267

*© 2001 Cancer Research Campaign*
